# A robust method for investigating thalamic white matter tracts after traumatic brain injury

**DOI:** 10.1016/j.neuroimage.2012.07.016

**Published:** 2012-11-01

**Authors:** Letizia Squarcina, Alessandra Bertoldo, Timothy E. Ham, Rolf Heckemann, David J. Sharp

**Affiliations:** a Department of Information Engineering, University of Padova, via Gradenigo 6/B, 35131 Padova, Italy; b Computational, Cognitive, and Clinical Neuroimaging Laboratory, Division of Brain Sciences, Imperial College London, Hammersmith Hospital Campus, Du Cane Road, London, W12 0NN, UK; c The Neurodis Foundation, CERMEP Imagerie du Vivant, 59 Boulevard Pinel, 69003 Lyon, France; d Division of Brain Sciences, Imperial College London, Hammersmith Hospital Campus, Du Cane Road, London, W12 0NN, UK

**Keywords:** TH, thalamus, ACCR, right anterior cingulate cortex, ACCL, left anterior cingulate cortex, IFGR, right inferior frontal gyrus, IFGL, left inferior frontal gyrus, SFGR, right superior frontal gyrus, SFGL, left superior frontal gyrus, SPLR, right superior parietal lobe, SPLL, left superior parietal lobe, STGR, right superior temporal gyrus, STGL, left superior temporal lobe, Diffusion tensor imaging, Tractography, Thalamus, Traumatic axonal injury

## Abstract

Damage to the structural connections of the thalamus is a frequent feature of traumatic brain injury (TBI) and can be a key factor in determining clinical outcome. Until recently it has been difficult to quantify the extent of this damage *in vivo*. Diffusion tensor imaging (DTI) provides a validated method to investigate traumatic axonal injury, and can be applied to quantify damage to thalamic connections. DTI can also be used to assess white matter tract structure using tractography, and this technique has been used to study thalamo-cortical connections in the healthy brain. However, the presence of white matter injury can cause failure of tractography algorithms. Here, we report a method for investigating thalamo-cortical connectivity that bypasses the need for individual tractography. We first created a template for a number of thalamo-cortical connections using probabilistic tractography performed in ten healthy subjects. This template for investigating white matter structure was validated by comparison with individual tractography in the same group, as well as in an independent control group (N = 11). We also evaluated two methods of masking tract location using the tract skeleton generated by tract based spatial statistics, and a cerebrospinal fluid mask. Voxel-wise estimates of fractional anisotropy derived from the template were more strongly correlated with individual tractography when both types of masking were used. The tract templates were then used to sample DTI measures from a group of TBI patients (N = 22), with direct comparison performed against probabilistic tractography in individual patients. Probabilistic tractography often failed to produce anatomically plausible tracts in TBI patients. Importantly, we show that this problem increases as tracts become more damaged, and leads to underestimation of the amount of traumatic axonal injury. In contrast, the tract template can be used in these cases, allowing a more accurate assessment of white matter damage. In summary, we propose a method suitable for assessing specific thalamo-cortical white matter connections after TBI that is robust to the presence of varying amounts of traumatic axonal injury, as well as highlighting the potential problems of applying tractography algorithms in patient populations.

## Highlights

► TBI produces significant damage to thalamo-cortical white matter connections. ► This damage disrupts probabilistic tractography in patients. ► The error associated with patient tractography increases with tract damage. ► A template approach allows more accurate estimation of tract damage after TBI.

## Introduction

Traumatic brain injury (TBI) is the commonest cause of death and disability in people under the age of 40 (Bruns and Hauser, 2003). Patients who survive are often left with cognitive and neuropsychiatric disorders that can result in long-lasting disability (Whitnall et al., 2006). Traumatic axonal injury (TAI) is a key pathological factor in the development of these problems (Adams et al., 2011; Inglese et al., 2005; Meythaler et al., 2001; Povlishock and Katz, 2005; Smith et al., 2003). Widespread TAI produces damage to white matter pathways, disrupting the functioning of distributed brain networks (Kinnunen et al., 2011; Mac Donald et al., 2007). The thalamus is a key node in many of these networks (Alkonyi et al., 2010, 2011; Behrens et al., 2003a), and damage to its structural connectivity is an important determinant of outcome after TBI ( Adams et al., 2000). Therefore, accurately quantifying TAI in thalamic connections is an important clinical goal, not least because the efficacy of novel treatments for TBI, such as deep brain stimulation, will probably depend upon whether thalamic connections are intact after TBI (Schiff et al., 2007).

Until recently it has been difficult to study thalamic connectivity in *vivo*. The development of diffusion tensor imaging (DTI) has provided a flexible means of investigating thalamo-cortical connections (Behrens et al., 2003a). Water diffusion within a structure can be measured using MRI (Basser and Pierpaoli, 1996; Beaulieu, 2002). The structure of axons constrains diffusion to occur predominantly in parallel to the main direction of the tract. This anisotropic diffusion pattern provides the basis for inferring white matter structure. Diffusion data is often analysed by fitting a tensor model to the acquired data. The tensor is then represented by its three eigenvectors (λ_1,_ λ_2,_ λ_3_) and corresponding eigenvalues. Fractional anisotropy (FA) describes the degree of anisotropy of diffusion, and mean diffusivity (MD) describes the magnitude of diffusion.

DTI can be used to delineate white matter tracts *in vivo*. A completely probabilistic approach for DTI tractography has been introduced by Behrens et al. (2003b, 2007)). Here, DTI data is analyzed within a Bayesian probabilistic framework and the noise is characterized statistically. A probability density function (PDF) associated with the direction of diffusion is calculated. The probability that a voxel is connected to another is computed after drawing a large number of samples from its PDF. Once a tract location has been estimated, this can be used to provide estimates of the amount of white matter damage within specific tracts (Kanaan et al., 2006; Glenn et al., 2003; Pagani et al., 2005; Xue et al., 1999).

Patients with white matter damage pose challenges to each of these methods (Hua et al., 2008; Singh et al., 2010). In the uninjured brain the standard approach is to perform tractography in individual space. However, when performed in patients, any ‘abnormalities’ in the tract structure can be difficult to interpret. There are a number of reasons for this. In addition to the physical absence of a tract, the procedure might fail if the amount of white matter damage in a tract is sufficiently large, as FA will often be low enough or the uncertainty high enough to impair performance of the tractography algorithm (Hua et al., 2008). This can result in parts of the tract appearing to be absent (Hashimoto et al., 2007; Rutgers, et al., 2008), or in an apparent reduction in the number of tract fibers compared to controls (Singh et al., 2010). Estimates of tract integrity derived from the resulting incomplete tractography might be misleading, and may even underestimate the amount of damage within a tract, if areas of severe damage have been excluded from the reconstructed tract.

The problems of performing tractography in patient groups have led to the use of tract atlases derived from control subjects and applied to patients. For example, Hua et al. (2008) used deterministic tractography to define the structure of a number of large white matter tracts in healthy controls, which were combined into average tracts. This information was used to study tract structure in an MS patient where tractography failed due to demyelination. This approach was then extended to study a group of MS patients (Reich et al., 2010), and was shown to allow diffusivity indices to be derived from parts of the white matter tract that are not detectable when tractography algorithms are applied directly on patient DTI data.

In this study, we investigated thalamo-cortical connectivity following TBI using DTI data. In contrast to Hua and colleagues we used probabilistic tractography in a group of young control subjects to infer the location of a number of thalamo-cortical connections. These were then combined across subjects to derive templates of average thalamo-cortical connections, which were used to investigate white matter structure along these tracts. To validate the approach, we determined various diffusion metrics on healthy subjects by applying the template, and compared them with values obtained using individual tractography. The advantages of taking the template approach in patients are illustrated by comparing template and individual tractography results. In contrast to previous work, we quantitatively compare measures of tract abnormality using the two approaches (Hua et al., 2008; Reich et al., 2010). Unlike previous approaches, our method is completely automatic and does not require user-defined parameter settings. We also address the partial volume and registration problems that have previously been highlighted (Reich et al., 2010) by using non-linear registration to improve the accuracy of transforming individual tracts into standard space, and by investigating the use of two masking techniques to reduce partial volume effects.

## Material and methods

### Subjects: demographics and clinical details

We recruited 22 TBI patients (age 39 +/− 11 years, 17 males, 5 females, demographics and clinical details reported in Table 1) and 21 age-matched healthy control subjects (age 35 +/− 12 years, 10 males, 11 females). Patients were investigated at least two months post injury (23 +/− 17 months), having been referred to their local TBI service because of persistent neurological problems. Based on the Mayo classification system for TBI severity (Malec et al., 2007) there were 18 moderate/severe, and 4 mild (probable) cases of TBI. This system integrates the duration of loss of consciousness, length of post-traumatic amnesia, lowest recorded Glasgow Coma Scale in the first 24 h, and initial neuroimaging results. Injuries were secondary to road traffic accidents (36%), falls (27%), assaults (27%), sport injuries (4.5%) and other causes (4.5%). Exclusion criteria were as follows: neurosurgery, except for invasive intracranial pressure monitoring (one patient); history of psychiatric or neurological illness prior to their head injury; history of significant previous TBI; pre-existing epilepsy; current or previous drug or alcohol abuse; contraindication to MRI. The study was approved by the Hammersmith, Queen Charlotte's and Chelsea Research Ethics Committee. All participants gave written informed consent.

### Clinical imaging

All patients had abnormalities on initial CT imaging and/or follow-up MRI. Standard T1 MRI was used to assess focal brain injury, and gradient echo imaging to identify microbleeds, which are a marker of diffuse axonal injury (DAI) (Scheid et al., 2003). A senior consultant neuroradiologist reviewed all MRI scans. At the time of the study 4 patients had residual evidence of contusions, 8 had microbleeds as demonstrated on gradient echo imaging and 1 had evidence of both. Contusions were mainly situated in the inferior parts of the frontal lobes, including the orbitofrontal cortex, and the temporal poles, in a typical lesion distribution for TBI patients (Gentry et al, 1988).

### MRI acquisition

MRI data were obtained using an Intera 3.0 Tesla MRI scanner (Philips Medical Systems, Best, The Netherlands) using Nova Dual gradients, an 8 channel phased array head coil, and sensitivity encoding (SENSE) with an under-sampling factor of 2. High-resolution images (T1-weighted MPRAGE) were acquired with the following acquisition parameters: matrix size 208 × 208; slice thickness = 1.2 mm, 0.94 mm × 0.94 mm in plane resolution, 150 slices; TR = 9.6 ms; TE = 4.5 ms; flip angle 8°. Diffusion-weighted volumes with gradients applied in 64 non-collinear directions were collected. The following parameters were used: 73 contiguous slices, slice thickness = 2 mm, field of view (FOV) 224 mm, matrix 128 × 128 (voxel size = 1.75 × 1.75 × 2 mm^3^), b value = 1000 s/mm^2^. Four images with no diffusion weighting (b = 0 s/mm^2^) were also acquired. At the time of acquisition all data was carefully checked for the impact of large movements, which produce obvious abnormalities in the raw data. Where this was the case the scan acquisition was repeated. In a small number of cases subjects were excluded from further analysis because of excess head movement.

### Data processing

**Fig. 1:**
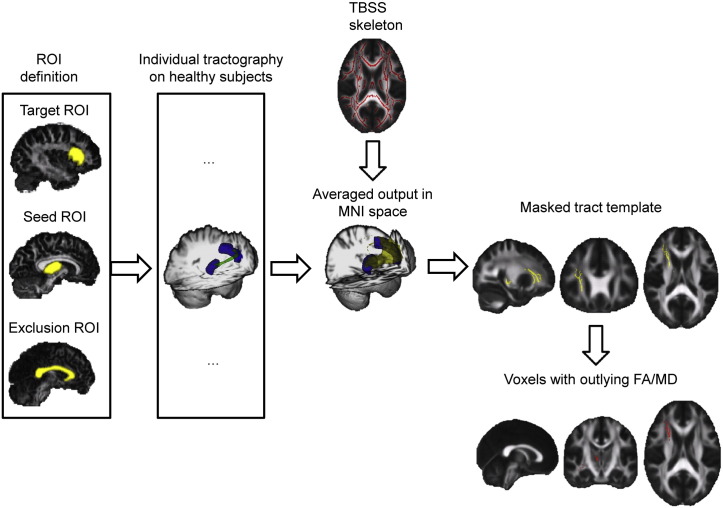
Overview of the analysis. Seed, target and exclusion ROIs for tractography are identified in subject space for all healthy controls. Individual tractography is run for all healthy subjects. The output is non-linearly transformed to MNI 1 mm^3^ standard space, averaged and binarized to create a mean white matter thalamo-cortical connectivity template. The maps are then masked with a TBSS white matter skeleton. Patients’ FA and MD values are compared to healthy mean FA and MD within the specified tracts, and voxels with abnormal values are counted.

A high level overview of the methodology used is provided in Fig. 1. Our approach comprised the following steps: 1) data preprocessing; 2) definition of anatomical regions-of-interest in the control group for use as start and end points for tractography; 3) probabilistic tractography in the control group; 4) generation of mean thalamo-cortical tract templates; 5) validation of the tract templates in two control groups; 6) investigation of white matter structure in the TBI group.

#### Diffusion tensor imaging preprocessing

To minimize distortion caused by eddy currents and small head motion, diffusion-weighted images were registered to the non-diffusion-weighted (b = 0) image by affine transformations. These were then brain-extracted using BET (Smith, 2002), part of the FSL image processing toolbox (Smith et al., 2004; Woolrich et al., 2009). Fractional anisotropy (FA) and mean diffusivity (MD) maps were generated using FDT in FSL (Behrens et al., 2003b, 2007).

#### Brain segmentation and region of interest definition

The MAPER (multi-atlas propagation with enhanced registration) procedure was used to generate ROIs for the start and target points for tractography (Heckemann et al., 2010). Anatomical ROIs were defined in native space from high-resolution T1 images. The approach has previously been shown to yield accurate segmentation results when compared with manual segmentation (Heckemann et al., 2006). It is robust in the presence of pathology (Heckemann et al., 2011) and yields reproducible results when used as a basis for estimating regional connectivity (Traynor et al., 2010). It minimizes the potential sampling error associated with mis-registration by propagating segmentation labels from multiple templates (Heckemann et al., 2006, 2010). Bilateral thalamic regions were defined to provide the starting point of tractography. A number of cortical regions were then defined to provide the termination points of thalamo-cortical tracts likely to be damaged by TBI. These were bilateral anterior cingulate cortices, superior frontal, inferior frontal, and superior temporal gyri, as well as superior parietal lobe regions. Detailed descriptions of the boundaries of these regions can be found in Hammers et al. (2003) and Gousias et al. (2008).

#### Probabilistic tractography

Probabilistic tractography was used to define thalamo-cortical connections in the control group (Behrens et al., 2003b, 2007). The left and right thalamic ROIs were used separately as starting points. Ipsilateral cortical regions provided the targets for tractography. To limit inter-hemispheric connections, a corpus callosum ROI obtained from the MAPER procedure was used as exclusion mask. Tractography was performed in DTI subject space. To propagate MAPER labels to DTI space, DTI b = 0 images were linearly registered to T1 images and the inverted transformation matrix was applied to the T1. The probabilistic tractography algorithm considered a maximum of two fibers per voxel. A curvature threshold of 0.2 was employed, with 5000 samples producing the estimated fiber distribution for each seed voxel. In this way ten tracts were generated for every subject. For each subject, connectivity values were divided by the total number of generated streamlines. In this way, the results are not biased by subjects that show higher values of connectivity, which can for example be caused by bigger starting or terminating ROIs in individual space.

#### Creating mean thalamo-cortical tracts

The tractography from 10 control subjects was then combined to create a template of mean thalamo-cortical tracts. Individual FA maps were non-linearly warped and registered to the 1 × 1 × 1 mm^3^ *FMRIB* MNI FA template using the FSL FNIRT tool (Andersson et al, 2008). The transformation matrix obtained was then applied to the individual tractography outputs. The connectivity maps obtained from tractography, which include all voxels with value greater than zero, were then warped to standard space. Voxel values were interpolated utilizing a nearest neighbor technique, to avoid smoothing effects that could alter the connectivity values. Thalamo-cortical tract templates were generated by first averaging the individual warped connectivity values in standard space, and then retaining only the voxels whose intensity exceeded a threshold set at the 95th percentile of the distribution of the connectivity values obtained from the averaging process. After thresholding the tracts were binarized. The resulting maps identified voxels with a high likelihood of falling within a tract of interest across all controls.

#### Comparing white matter integrity estimates based on different tract estimations

The validity of using a tract template to sample white matter integrity measures was assessed in the control subjects. We reasoned that the maps obtained using the mean tract template should be similar to those obtained using individual tractography. To verify this, we compared mean FA values derived from the tracts generated by both individual tractography and the tract templates. To ensure comparability between each of the individual templates and the normalized template, we modified the binarization threshold for each individual connectivity map, such that each resulting individual template was volume-equivalent to the normalized template.

A key requirement for our method is that the tracts defined in individual space can be registered accurately into standard space. Any inaccuracy would result in partial volume errors when DTI measures are calculated, particularly around the edge of tracts (Smith et al., 2006). To minimize this problem we employed non-linear registration algorithms (Andersson et al, 2008). We also investigated the benefits of masking areas of the white matter that are prone to partial volume effects. We used two types of masking. First, the white matter skeleton produced by tract-based spatial statistics (TBSS, Smith et al, 2006) was used as a mask. This skeleton defines the central points of large white matter tracts, and thus provides a robust method of focusing the voxel-wise analysis on locations where the risk of partial volume effects is low. We generated the skeleton considering the 10 subjects from which the template was generated, then we considered the other 11 healthy subjects, and finally all 21 healthy controls. As can be seen in Supplementary Fig. 1, very similar results were obtained considering the different groups. Second, a CSF mask produced by FAST (Zhang et al, 2001) and the grey matter masks of start and target regions were used to further limit the extent of the tract template. DTI measures from the individual tractography and tract template approaches were then compared in three configurations: 1) no masking; 2) white matter ‘skeleton’ masking; and 3) ‘skeleton’ + CSF + ROIs masking. Spearman's correlation statistics were used for mean MD and FA values. We performed the same set of analyses on a further group of 11 control subjects, who had not been used to generate the template tracts. This was done to test whether the tract templates could be applied outside the original group.

#### Tractography in the patient group

We then directly tested whether individual tractography in the patient group was problematic. Tractography was performed using the same procedure as for controls. We calculated diffusion measures for the resulting tracts, and we also determined estimates of dispersion of the principal and second diffusion direction at each voxel. This provides a measure of the noise, and hence the uncertainty, of the estimation of the diffusion direction when sampling from its probability density function. We hypothesized that dispersion would be increased in patients as a result of the white matter damage caused by TBI.

#### Investigation of white matter damage in the patients

FA and MD maps of patients were non-linearly registered to MNI space using FNIRT (Andersson et al, 2008). FA and MD values were then sampled using the tract templates defined in the control group. Building on the results of the experiment described in above, both masking techniques (TBSS skeleton and CSF masking) were used in the patient analyses. We predicted that increasing white matter damage would result in a greater discrepancy between the two approaches, because individual tractography would be increasingly prone to error as damage increased. We tested this by comparing estimates of damage from the template approach with the discrepancy in estimates between the two approaches. The significance of these correlations was tested using Spearman's correlation coefficients with a significance level of *P* < 0.05.

Tract damage was estimated for all voxels in each tract. This was estimated by calculating a Z score based on the comparison of DTI measures between patients and all 21 age-matched controls. For both FA and MD, voxels with a Z > 3 were considered outliers. This was taken to indicate a high likelihood of white matter damage at that particular voxel. Similar calculations were made for the individual tractography approach, allowing a comparison of the estimates of white matter damage produced by the two approaches.

## Results

### Comparing template and individual tractography approaches

**Fig. 2:**
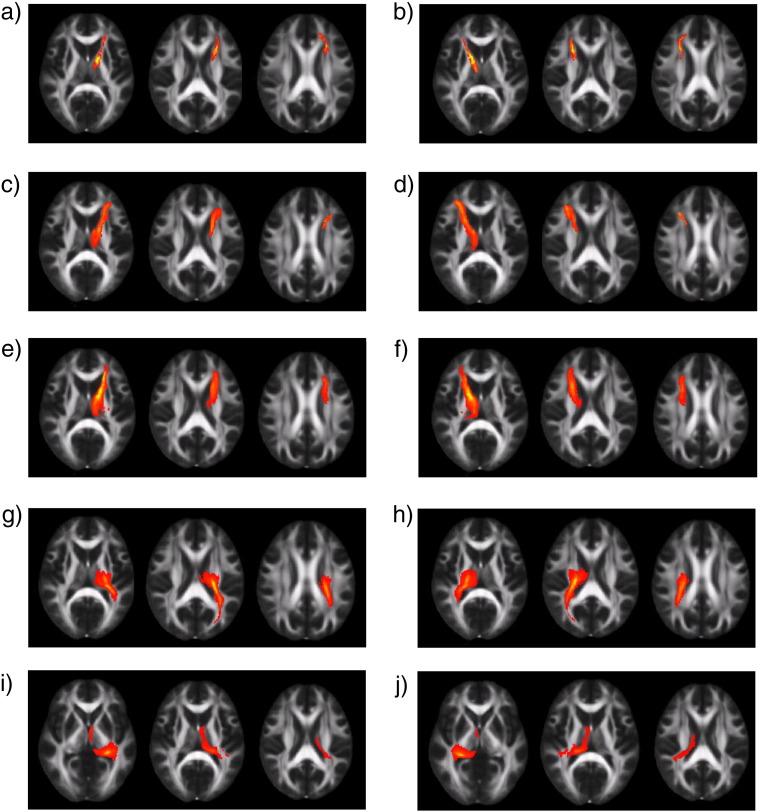
FA maps in standard space with the thresholded tract overlaid (red-yellow voxels: yellow voxels have higher probability). The threshold was set at the 95th percentile of the distribution of the connectivity values. a) left thalamus–left anterior cingulate gyrus; b) right thalamus–right anterior cingulate gyrus; c) left thalamus–left inferior frontal gyrus; d) right thalamus–right inferior frontal gyrus; e) left thalamus–left superior frontal gyrus; f) right thalamus–right superior frontal gyrus; g) left thalamus–left superior parietal lobe; h) right thalamus–right superior parietal lobe; i) left thalamus–left superior temporal gyrus; j) right thalamus–right superior temporal gyrus.

Ten thalamo-cortical tracts tract templates were generated (Fig. 2), and used to estimate FA and MD in individual DTI data sets. These estimates were then compared with the estimates extracted from probabilistic tractography performed in each individual. Visual inspection revealed a good correspondence in the spatial location using both approaches to tract generation. We first extracted DTI measures from the group of subjects used to define the templates (N = 10). In the case of FA, only 2 tracts showed a significant correlation between the two approaches when no masking was used. In contrast, 7 tracts showed significant correlation when TBSS skeleton masking was used to constrain the search area, and also when additional gray matter and CSF masking were used. For MD, only 4 tracts were significantly correlated without applying masking, whereas all tracts were correlated when either masking technique was applied.

To test how generalizable the relationship was we performed the same analysis in a second group of controls not used to create the template (N = 11). Here, 5 tracts showed significant correlation for FA without masking. Masking increased the number of correlated tracts for FA to 7 with both TBSS skeleton and CSF/gray matter masking. For MD, 7 tracts correlated significantly without masking. With the two masking approaches, all tracts were significantly correlated (Table 2). These results demonstrate that focusing the analysis of white matter to regions at the center of each tract produces results that converge between the approaches. This supports the proposition that a mean tract template can be used in most cases to robustly sample DTI metrics from large white matter tracts, when the analysis is focused on the central points of tracts. In addition, they show that this approach can be generalized to subjects not used to define the original tract templates.

### Investigation of white matter damage in patients

In the TBI patients we investigated the extent of white matter tract damage by calculating the percentage of voxels with abnormal MD and FA values. Given the results of the control analyses, we used TBSS skeleton and CSF/gray matter masking for these analyses. Abnormality in white matter of patients is detected using the template when considering the number of voxels with high MD in respect to healthy controls. We computed the number of outlying voxels in patients and in the 11 healthy controls not involved in the creation of the template. The number of outlying voxels computed in patients is consistently higher than in healthy controls (Wilcoxon rank‐sum test, *P* = 0.05), as can be seen in Supplementary Fig. 2.

#### White matter damage following TBI disrupts probabilistic tractography

**Fig. 3:**
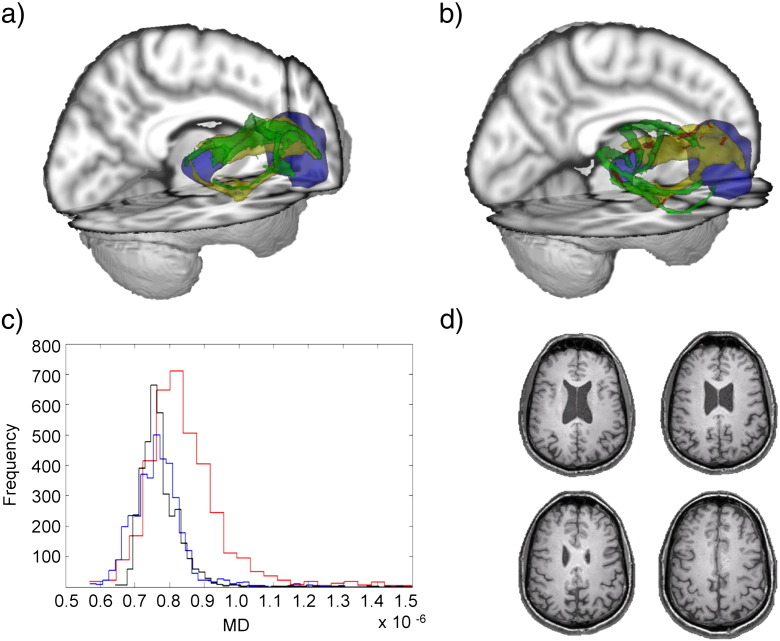
3D representations from two patients of the tract connecting the right thalamus to the right anterior cingulate cortex (ACC). Thalamic and ACC regions are shown in blue, along with the average tract template (yellow), individual patient tractography (green), and abnormal voxels (red, defined as MD > MD_mean_ +/− 3sd). (a) A patient with a small percentage of damaged voxels (1.17%, Patient 1). (b) A patient with a large percentage of damaged voxels (28.81%, Patient 5). (c) Histogram of MD values. Black: mean atlas MD. Blue: MD values for patient (a). Red: MD values for patient depicted in (b). (d) Four T1 slices for the patient illustrated in (b), showing an absence of major white matter structural damage.

**Fig. 4:**
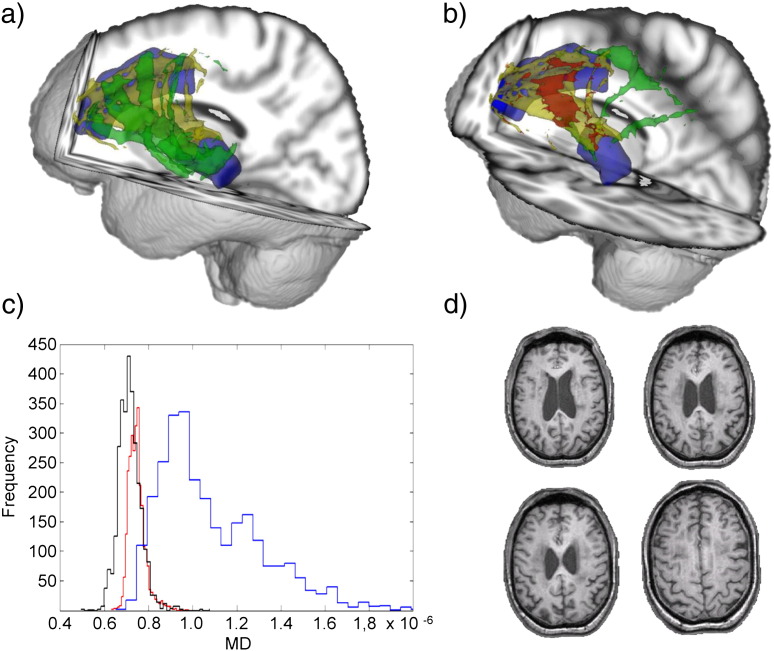
3D representation from two patients of the tract connecting the left thalamus to the left inferior frontal gyrus. Thalamic and inferior frontal gyrus regions are shown in blue, along with tract and voxel abnormalities as in Fig. 3. (a) A patient with a small percentage of damaged voxels (0.69%, Patient 1). (b) A patient with a large percentage of damaged voxels (47.96%, Patient 6). (c) Histogram of MD values in the same tract depicted above. Black: mean atlas MD. Blue: MD values for Patient 1. Red: MD values for Patient 6. (d) Four T1 slices from the patient illustrated in (b), showing an absence of major white matter structural damage.

The limitations of probabilistic tractography performed on individual patients’ DTI data are illustrated by considering patients with high and low levels of tract damage. For example, in a patient with a relatively normal tract connecting the right thalamus to the right ACC (Patient 1: 1.17% voxels with abnormal MD, 0% abnormal FA), the result of individual tractography was similar to the result based on the template approach (Fig. 3a). In contrast, in a patient with a large amount of damage to this tract (Patient 5: 28.81% voxels with abnormal MD, 7.19% abnormal FA), individual tractography produced a highly atypical result, with voxels extending far outside the probable anatomical location of this tract (Fig. 3b). Since there were no gross abnormalities visible on standard structural imaging in this region, this is likely to be a spurious result. The most likely explanation is that assumptions built into the tractography algorithm about the white matter structure are violated in the presence of white matter damage. A similar finding is illustrated for the tract connecting the left thalamus to the left inferior frontal gyrus tract (Fig. 4). The output of individual tractography corresponds with the template location for a patient with little damage (Patient 1: 0.69% voxels with abnormal MD, 0% abnormal FA), whereas, in a patient with very abnormal tract white matter (Patient 6: 47.96% of voxels with abnormal MD, 27.35% abnormal FA), the individual tractography failed almost completely to reproduce the location of the mean tract (Fig. 4b).

#### Probabilistic tractography underestimates white matter damage

**Fig. 5:**
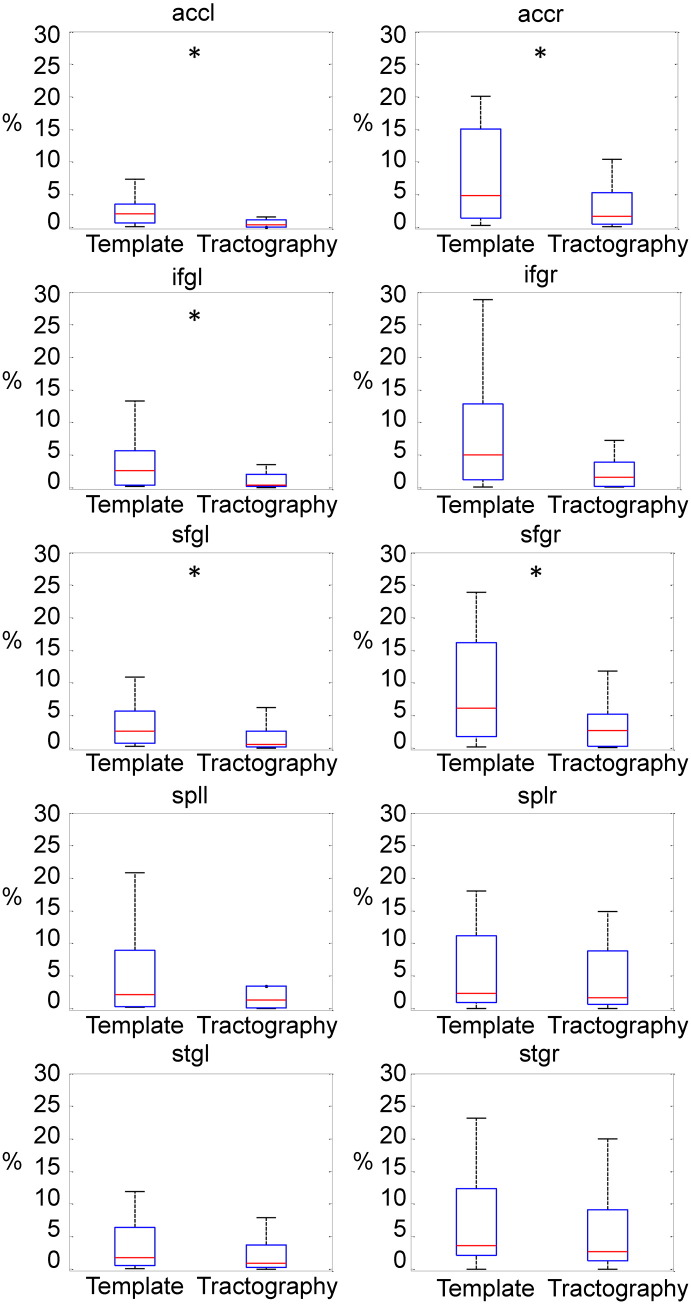
Individual tractography underestimates the amount of white matter damage following TBI. Boxplots showing the mean percentage of voxels with MD > (mean MD + 3sd) in patients, calculated using the thalamo-cortical template (left) and using direct tractography applied to each individual (right). ANOVA demonstrated a main effect of analysis type across all the tracts. For illustration purposes the tracts where the estimated number of damaged voxels is significantly higher than the thalamo-cortical template are marked with an asterisk (Wilcoxon rank-sum test, *P* < 0.05),.

With the template approach, the estimated number of voxels with abnormal MD values in TBI patients was consistently higher than with the individual tractography approach (see Fig. 5). For MD, ANOVA showed a main effect of the analysis technique (F(1,20) = 6.89, *P* < 0.05), with higher MD values for the template approach. There was no significant difference in the FA estimates between the approaches.

**Fig. 6:**
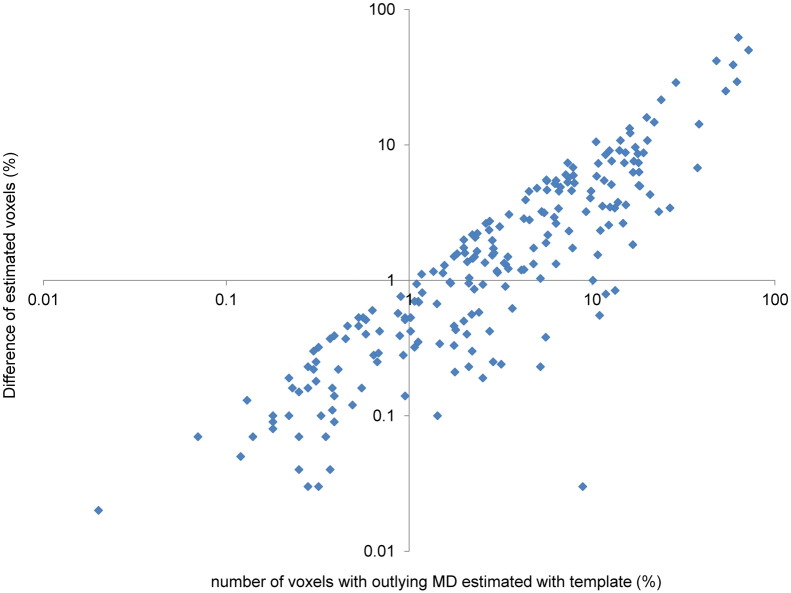
The error associated with individual tractography increases with the amount of tract damage. Plot of the estimated number of damaged voxels with the thalamo-cortical template against the difference between the number of estimated damaged voxels obtained using the two methods (Spearman ρ = 0.89, *P* < 0.001). The quantities are shown on logarithmic scales, for clarity.

The difference between the two approaches was a function of the amount of tract damage. A greater underestimation of voxels with abnormal MD values was found in tracts with more damage. This is demonstrated by the striking correlation between abnormal tract MD derived using the template approach, and the discrepancy between MD values derived from both techniques (ρ = 0.89, *P* < 0.001) (Fig. 6). This clearly demonstrates that as tract damage increases, the error in estimating white matter structure using individual tractography increases. In combination with the spurious results of tractography when visually inspected, these results demonstrate that the discrepancy between the techniques is due to underestimation of white matter damage by the probabilistic tractography approach, rather than overestimation by the template approach.

#### Uncertainty in the direction of diffusion (dispersion) is elevated following TBI

**Fig. 7:**
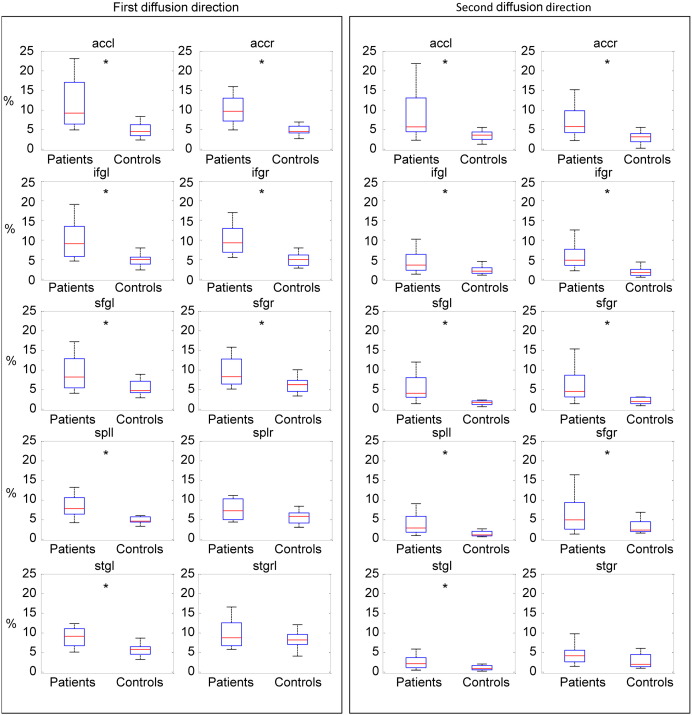
Box plots showing the mean percentage of voxels with dispersion higher than the mean dispersion in healthy subjects + 3SD for the principal and second diffusion directions. Tracts with a significantly higher dispersion for TBI patients than controls are marked with an asterisk (Wilcoxon rank-sum test, *P* < 0.05).

We next investigated dispersion in the patient and control groups. This is the key determinant of the probabilistic tractography algorithm as a high dispersion results in a high degree of uncertainty in the direction of tracking and potentially a failure of tractography. Patients in general had a greater dispersion for most of the tracts than controls, providing an explanation of the failure of tractography (Fig. 7).

## Discussion

Thalamic dysfunction is an important determinant of clinical outcome after TBI (Adams et al., 2011). Interventions aimed at improving thalamic function, such as deep brain stimulation, provide important new potential treatments (Schiff et al, 2007). Their efficacy will depend upon the extent of structural damage to thalamic connections. Therefore, the ability to accurately quantify the amount of such damage is likely to become clinically important. Thalamo-cortical connections can be defined in the healthy brain with tractography analysis of DTI data. This approach has been used directly in TBI patients to investigate the location and structure of tract damage (Rutgers et al, 2008; Nakayama et al, 2006; Wang et al, 2011). However, the technique is not robust to highly damaged white matter (Hua et al, 2008). Here we show that TBI frequently results in significant damage to thalamo-cortical connections, and that this damage disrupts probabilistic tractography. When significant damage is present, the tract estimations produced are often anatomically implausible. This is the result of the high levels of uncertainty produced when the principal directions of diffusion are estimated. As a result, DTI estimates of white matter damage extracted from tracts produced directly in TBI patients are difficult to interpret. Importantly, the error associated with this approach increases with the amount of tract damage, which is particularly problematic for patient studies. We propose an alternative method for investigating damage to thalamo-cortical tracts after TBI. This is based on the generation of templates for thalamo-cortical tracts defined in healthy controls, which are then used to sample DTI measures from anatomical regions of interest in TBI patients. We show this method to be suitable for studying the integrity of specific white matter tracts after TBI, and to be robust to the presence of highly abnormal FA.

DTI is increasingly used to study the pathological effects of TBI (Sharp and Ham, 2011; Sharp et al, 2011; Mac Donald et al., 2007; Kinnunen et al., 2011; Sidaros et al., 2008). The manual definition of regions of interest in individual DTI space provides a robust and practical approach for investigating white matter regions when they can easily be delineated and subject numbers are small. (Mac Donald et al., 2007; Sidaros et al, 2008; Niogi et al, 2008). In addition, methods such as tract based spatial statistics (TBSS) provide an alternative approach that allows the voxelwise assessment of all parts of large white matter tracts in an automated way (Smith et al, 2006; Kinnunen et al, 2011). However, it may be desirable to study the structure of particular white matter tracts, for example to assess the impact of damage to specific brain networks (Bonnelle et al, 2011, 2012). In this situation manual approaches are problematic because: a) it may not be possible to define the location of the tracts of interest by eye; and b) it may be impractical to manually define tracts in large patient groups. In contrast, methods such as TBSS provide whole-brain voxelwise measures, but do not separate the output into specific white matter tracts. We show how the use of mean thalamo-cortical tract templates coupled with careful non-linear registration and masking of individual DTI data may solve these problems. Tract locations are defined in healthy controls and then applied as regions of interest in TBI patients allowing the damage to specific tracts to be quantified.

Our approach builds on previous work in patients with multiple sclerosis, where demyelinating plaques are associated with low FA values (Hua et al, 2008; Reich et al, 2010). The use of white matter tract templates has been shown to provide a solution for some of the problems of performing tractography directly in this patient group. Hua et al. (2008) used deterministic tractography to generate probability maps for 11 large white matter tracts using DTI data from healthy controls as the starting point. They showed strong correspondence between DTI measures extracted from these maps and from tracts produced in individuals, a finding which our results corroborate. Hua and colleagues went on to perform tractography in a single patient with multiple sclerosis and found that regions of reduced FA associated with demyelination disrupted tract reconstruction. These results have subsequently been confirmed in a larger study of multiple sclerosis patients (Reich et al., 2010), and provide the motivation for investigating whether mean tract templates can be used to investigate white matter damage in other patient groups.

There have also been important recent advances in the use of *a priori* tract template information to improve the performance of tractography in the healthy brain. Zhang et al. (2010) have recently defined a set of manually defined templates. These provide information about the expected trajectory of tracts, which can be used to guide tractography. This allows a semi-automated approach to deterministic tractography, and provides benefits in terms the reproducibility of generated tracts. The approach also makes it easier to define some tracts, such as thalamo-cortical connections, that were previously difficult to study using deterministic tractography. Tozer et al. (2012) provide another example of the use of templates for tractography. They generated a number of white matter tracts in standard space also using deterministic tractography, and after averaging the acquired DTI data propagated tracts into the adjacent gray matter. This allows gray matter connected to specific tracts to be clearly defined, providing a way of investigating the effects of white matter pathology on cortical structure and function.

Our findings extend these studies in a number of ways. Firstly, in a comparatively large group of TBI patients we show that individual tractography in TBI patients has similar limitations to those seen in patients with multiple sclerosis. In addition, we study thalamo-cortical connectivity, focusing our analysis on ten distinct tracts that are frequently damaged as a result of TBI. These tracts have been difficult to study using deterministic tractography, which motivates another important difference to previous work, the use of probabilistic tractography. This offers some advantages over deterministic tractography, particularly when the tracts of interest cannot be located anatomically with any certainty. Probabilistic tractography is a more automated means of tract location, and this can be particularly beneficial when investigating the connectivity of subcortical structures. Importantly, we show that probabilistic tractography is subject to the same disruptive factors as is deterministic tractography, and that probability templates generated using probabilistic tractography in healthy controls can be used to circumvent this problem, by generating mean tract templates with which to sample tract structure following TBI.

We also show the benefit of using masking to focus the analysis on the central points of white matter tracts, which greatly reduces the problem of partial volume error. Accurate registration between standard and individual DTI spaces is required for our approach to work well. This is particularly important because the sampling of DTI measures is sensitive to partial volume effects. If a voxel is within a tract but close to its edge, a small error in registration may result in gray matter or CSF being sampled instead of the tract's white matter. We addressed this issue using a combination of highly accurate nonlinear registration and masking with the white matter ‘skeleton’ produced by tract based spatial statistics (TBSS, Smith et al, 2006). We combined the non-linearly registered mean tract templates with individual white matter skeletons to produce a map of the central part of each tract. This has the effect of excluding voxels towards the edge of a tract, making the technique more robust to partial volume effects. When this masking was combined with an exclusion mask for CSF and gray matter, the DTI results produced by the mean template approach correlated for all tracts with those produced by individual tractography. In the absence of a gold standard method for defining the location of tracts *in vivo*, this result supports the validity of our approach.

We also demonstrate that performing probabilistic tractography directly in patients and then sampling white matter structure from the resulting tract appears to result in underestimation of the amount of damage to the tract. This is likely to be due to a failure of the tractography algorithm to track through areas of severe damage, which are associated with low FA. As a result these are erroneously excluded from the resulting tract and so the overall estimate of FA is artificially elevated. This is particularly problematic because the error becomes larger with increasing tract damage. The reliability of the technique is thus inversely proportional to the amount of damage in the tract. The use of mean tract templates eliminates this serious problem, because the generation of the spatial location of the tract is not a function of the tract damage in the individual patient.

There are some potential limitations in the current study that should be considered. Firstly, although we used standard DTI pre-processing to achieve good quality registration, we did not include the use of a field map to additionally correct for the influence of susceptibility distortions. This does not appear to be a major issue, as careful manual checking suggested that good quality registration was achieved, even in areas where distortion might be expected such as the orbitofrontal cortex. Secondly, our use of the white matter skeleton focused the analysis on the centre of large white matter tracts. This approach could potentially miss pathology at the edges of tracts, or in the small branches of white matter tracts that are not assessed. This possible loss of sensitivity has to be balanced against the fact that our approach is more robust to small errors in registration and partial volume effects, which can be very problematic for the assessment of DTI metrics, particularly in patient populations. Thirdly, we used standard approaches to probabilistic tractography, and it is possible that advances in diffusion imaging such as HARDI might be better able to deal with white matter pathology in patient populations (Descoteaux et al., 2009). However, these advanced techniques are often difficult to apply in a clinical setting, because of long scan acquisition times. As a result, our findings are applicable to analysis of the type of data that is starting to be acquired as part of standardized clinical assessment.

### Conclusions

In summary, we show that probabilistic tractography that is performed directly in TBI patients can produce spurious results. When the resulting tracts are used to estimate the amount of tract damage, errors are produced that increase in magnitude with the severity of the underlying damage to the tract. We propose a technique that eliminates this problem. Tract templates are generated from a group of healthy controls, propagated to patient images using accurate non-linear registration, and masked to focus the analysis on the central points of large white matter tracts. The proposed analysis yields superior results, and could be applied in other patient groups where systematic differences in FA are likely to also compromise any tractography applied in individuals.

The following are the supplementary data related to this article.

Supplementary Fig. 1 Skeletons obtained using the 10 subjects from which the template is obtained (row a), the other 11 healthy subjects (row b) and the whole healthy population (c) in three slices in standard space. The skeletons are overlaid in different colors (row d), depicted in yellow, green and blue respectively. The voxels common to all three skeletons are depicted in red.

Supplementary Fig. 2 Boxplots showing the mean percentage of voxels with MD > (mean MD + 3 sd) in patients and in controls (11 subjects unrelated to the template creation process), for all the 10 tracts considered in this study. The percentages of outlying voxels are consistently smaller for healthy controls, as is to be expected (Wilcoxon rank‐sum test, *P* = 0.05).

## Figures and Tables

**Table 1:** Demographic and clinical data for patients.

Subject	Sex	Age	Cause of TBI	Initial GCS	Severity
1	M	25	Assault	13	Mod/severe
2	M	18	Fall	15	Mod/severe
3	M	23	Assault	15	Mod/severe
4	M	54	Fall	Not known	Mod/severe
5	M	37	Fall	4	Mod/severe
6	M	56	Fall	3	Mod/severe
7	F	50	RTA	3	Mod/severe
8	F	41	Assault	Not known	Mod/severe
9	M	34	Assault	Not known	Mod/severe
10	M	34	RTA	5	Mod/severe
11	F	49	RTA	Not known	Mod/severe
12	M	47	RTA	13	Moderate
13	M	47	Assault	Not known	Mod/severe
14	M	36	Assault	Not known	Mod/severe
15	M	23	Incidental blow	15	Mild
16	M	53	RTA	4	Mod/severe
17	M	29	RTA	6	Mod/severe
18	F	33	Fall	Not known	Mod/severe
19	M	42	Sport injury	14	Mild
20	F	24	Fall	15	Severe
21	M	49	RTA	Not known	Severe
22	M	47	RTA	14	Mild

**Table 2:** Correlations between FA and MD values of tracts defined using tractography applied to each individual and using the thalamo-cortical template. Values are calculated for the subjects used to generate the template (template sources) and for unrelated control subjects (non-sources). The correlations have been calculated either: a) without masking of the tracts (none); b) using masking with the TBSS skeleton (skel); or c) using this skeleton plus a mask of gray matter (GM) and cerebro-spinal fluid (CSF). The minimum, median and maximum values of correlation are reported along with the number of tracts showing a significant correlation between approaches.

Measure	Mask	Template sources (n = 10)				Non-sources (n = 11)			
		n sig.	Min	Median	Max	n sig.	Min	Median	Max
FA	None	2	0.88	0.88	0.87	5	0.72	0.78	0.82
Skeleton	7	0.6	0.81	0.96	7	0.71	0.83	0.91
Skel + GM + CSF	7	0.84	0.89	0.94	7	0.68	0.76	0.98
MD	None	4	0.66	0.69	0.73	7	0.63	0.73	0.88
Skeleton	10	0.85	0.90	0.97	10	0.65	0.86	0.98
Skel + GM + CSF	10	0.61	0.88	0.9	10	0.75	0.92	0.97

## References

[bb0005] Adams J.H., Graham D.I., Jennett B. (2000). The neuropathology of the vegetative state after an acute brain insult. Brain.

[bb0010] Adams J.H., Jennett B., Murray L.S., Teasdale G.M., Gennarelli T.A., Graham D.I. (2011). Neuropathological findings in disabled survivors of a head injury. J. Neurotrauma.

[bb0015] Alkonyi B., Chugani H.T., Behen M., Halverson S., Helder E., Makki M.I., Juhász C. (2010). The role of the thalamus in neuro-cognitive dysfunction in early unilateral hemispheric injury: a multimodality imaging study of children with Sturge–Weber syndrome. Eur. J. Paediatr. Neurol..

[bb0020] Alkonyi B., Juhász C., Muzik O., Behen M.E., Jeong J.W., Chugani H.T. (2011). Thalamocortical connectivity in healthy children: asymmetries and robust developmental changes between ages 8 and 17 years. AJNR Am J Neuroradiol.

[bb0025] Andersson J.L., Smith S.M., Jenkinson M. (2008). FNIRT–FMRIB's non-linear image registration tool. In: 14th Annual Meeting of the Organization for Human Brain Mapping; 15 June - 18 June 2008.

[bb0030] Basser P.J., Pierpaoli C. (1996). Microstructural and physiological features of tissues elucidated by quantitative-diffusion-tensor MRI. J Magn Reson. B.

[bb0035] Beaulieu C. (2002). The basis of anisotropic water diffusion in the nervous system—a technical review. NMR Biomed..

[bb0040] Behrens T.E., Berg H.J., Jbabdi S., Rushworth M.F., Woolrich M.W. (2007). Probabilistic diffusion tractography with multiple fibre orientations: what can we gain?. NeuroImage.

[bb0045] Behrens T.E., Johansen-Berg H., Woolrich M.W., Smith S.M., Wheeler-Kingshott C.A., Boulby P.A., Barker G.J., Sillery E.L., Sheehan K., Ciccarelli O., Thompson A.J., Brady J.M., Matthews P.M. (2003). Non-invasive mapping of connections between human thalamus and cortex using diffusion imaging. Nat. Neurosci..

[bb0050] Behrens T.E., Woolrich M.W., Jenkinson M., Johansen-Berg H., Nunes R.G., Clare S., Matthews P.M., Brady J.M., Smith S.M. (2003). Characterization and propagation of uncertainty in diffusion-weighted MR imaging. Magn. Reson. Med..

[bb0055] Bonnelle V., Ham T., Leech R., Kinnunen K.M., Mehta M.A., Greenwood R.J., Sharp D.J. (2012). Salience network integrity predicts default mode network function after traumatic brain injury. PNAS.

[bb0060] Bonnelle V., Leech R., Kinnunen K.M., Ham T.E., Beckmann C.F., De Boissezon X., Greenwood R.J., Sharp D.J. (2011). Default mode network connectivity predicts sustained attention deficits after traumatic brain injury. J. Neurosci..

[bb0065] Bruns J., Hauser W.A. (2003). The epidemiology of traumatic brain injury: a review. Epilepsia.

[bb0070] Descoteaux M., Deriche R., Knösche T.R., Anwander A. (2009). Deterministic and probabilistic tractography based on complex fibre orientation distributions. IEEE Trans. Med. Imaging.

[bb0075] Gentry L.R., Godersky J.C., Thompson B. (1988). MR imaging of head trauma: review of the distribution and radiopathologic features of traumatic lesions. AJR Am. J. Roentgenol..

[bb0080] Glenn O.A., Henry R.G., Berman J.I., Chang P.C., Miller S.P., Vigneron D.B., Barkovich A.J. (2003). DTI-based three-dimensional tractography detects differences in the pyramidal tracts of infants and children with congenital hemiparesis. J. Magn. Reson. Imaging.

[bb0085] Gousias I.S., Rueckert D., Heckemann R.A., Dyet L.E., Boardman J.P., Edwards A.D., Hammers A. (2008). Automatic segmentation of brain MRIs of 2-year-olds into 83 regions of interest. NeuroImage.

[bb0090] Hammers A., Allom R., Koepp M.J., Free S.L., Myers R., Lemieux L., Mitchell T.N., Brooks D.J., Duncan J.S. (2003). Three-dimensional maximum probability atlas of the human brain, with particular reference to the temporal lobe. Hum. Brain Mapp..

[bb0095] Hashimoto K., Okumura A., Shinoda J., Abo M., Nakamura T. (2007). Tensor magnetic resonance imaging in a case of mild traumatic brain injury with lowered verbal intelligence quotient. J. Rehabil. Med..

[bb0100] Heckemann R.A., Hajnal J.V., Aljabar P., Rueckert D., Hammers A. (2006). Automatic anatomical brain MRI segmentation combining label propagation and decision fusion. NeuroImage.

[bb0105] Heckemann R.A., Keihaninejad S., Aljabar P., Gray K.R., Nielsen C., Rueckert D., Hajnal J.V., Hammers A. (2011). Automatic morphometry in Alzheimer's disease and mild cognitive impairment. NeuroImage.

[bb0110] Heckemann R.A., Keihaninejad S., Aljabar P., Rueckert D., Hajnal J.V., Hammers A. (2010). Improving intersubject image registration using tissue-class information benefits robustness and accuracy of multi-atlas based anatomical segmentation. NeuroImage.

[bb0115] Hua K., Zhang J., Wakana S., Jiang H., Li X., Reich D.S., Calabresi P.A., Pekar J.J., van Zijl P.C., Mori S. (2008). Tract probability maps in stereotaxic spaces: analyses of white matter anatomy and tract-specific quantification. NeuroImage.

[bb0120] Inglese M., Makani S., Johnson G., Cohen B.A., Silver J.A., Gonen O., Grossman R.I. (2005). Diffuse axonal injury in mild traumatic brain injury: a diffusion tensor imaging study. J. Neurosurg..

[bb0125] Kanaan R.A., Shergill S.S., Barker G.J., Catani M., Ng V.W., Howard R., McGuire P.K., Jones D.K. (2006). Tract-specific anisotropy measurements in diffusion tensor imaging. Psychiatry Res..

[bb0130] Kinnunen K.M., Greenwood R., Powell J.H., Leech R., Hawkins P.C., Bonnelle V., Patel M.C., Counsell S.J., Sharp D.J. (2011). White matter damage and cognitive impairment after traumatic brain injury. Brain.

[bb0135] Mac Donald C.L., Dikranian K., Bayly P., Holtzman D., Brody D. (2007). Diffusion tensor imaging reliably detects experimental traumatic axonal injury and indicates approximate time of injury. J. Neurosci..

[bb0140] Malec J.F., Brown A.W., Leibson C.L., Flaada J.T., Mandrekar J.N., Diehl N.N., Perkins P.K. (2007). The mayo classification system for traumatic brain injury severity. J. Neurotrauma.

[bb0145] Meythaler J.M., Peduzzi J.D., Eleftheriou E., Novack T.A. (2001). Current concepts: diffuse axonal injury-associated traumatic brain injury. Arch. Phys. Med. Rehabil..

[bb0150] Nakayama N., Okumura A., Shinoda J., Yasokawa Y.T., Miwa K., Yoshimura S.I., Iwama T. (2006). Evidence for white matter disruption in traumatic brain injury without macroscopic lesions. J. Neurol. Neurosurg. Psychiatry.

[bb0155] Niogi S.N., Mukherjee P., Ghajar J., Johnson C., Kolster R.A., Sarkar R., Lee H., Meeker M., Zimmerman R.D., Manley G.T., McCandliss B.D. (2008). Extent of microstructural white matter injury in postconcussive syndrome correlates with impaired cognitive reaction time: a 3T diffusion tensor imaging study of mild traumatic brain injury. AJNR. Am. J. Neuroradiol..

[bb0160] Pagani E., Filippi M., Rocca M.A., Horsfield M.A. (2005). A method for obtaining tract-specific diffusion tensor MRI measurements in the presence of disease: application to patients with clinically isolated syndromes suggestive of multiple sclerosis. NeuroImage.

[bb0165] Povlishock J.T., Katz D.I. (2005). Update of neuropathology and neurological recovery after traumatic brain injury. J Head Trauma Rehabil..

[bb0170] Reich D.S., Ozturk A., Calabresi P.A., Mori S. (2010). Automated vs. conventional tractography in multiple sclerosis: variability and correlation with disability. NeuroImage.

[bb0175] Rutgers D.R., Fillard P., Paradot G., Tadié M., Lasjaunias P., Ducreux D. (2008). Diffusion tensor imaging characteristics of the corpus callosum in mild, moderate, and severe traumatic brain injury. AJNR Am. J. Neuroradiol..

[bb0180] Sharp D.J., Beckmann C.F., Greenwood R., Kinnunen K.M., Bonnelle V., De Boissezon X., Powell J.H., Counsell S.J., Patel M.C., Leech R. (2011). Default mode network functional and structural connectivity after traumatic brain injury. Brain.

[bb0185] Sharp D.J., Ham T.E. (2011). Investigating white matter injury after mild traumatic brain injury. Curr. Opin. Neurol..

[bb0190] Scheid R., Preul C., Gruber O., Wiggins C., von Cramon D.Y. (2003). Diffuse axonal injury associated with chronic traumatic brain injury: evidence from T2*-weighted gradient-echo imaging at 3 T. AJNR Am. J. Neuroradiol..

[bb0195] Schiff N.D., Giacino J.T., Kalmar K., Victor J.D., Baker K., Gerber M., Fritz B., Eisenberg B., Biondi T., O'Connor J., Kobylarz E.J., Farris S., Machado A., McCagg C., Plum F., Fins J.J., Rezai A.R. (2007). Behavioural improvements with thalamic stimulation after severe traumatic brain injury. Nature.

[bb0200] Sidaros A., Engberg A.W., Sidaros K., Liptrot M.G., Herning M., Petersen P., Paulson O.B., Jernigan T.L., Rostrup E. (2008). Diffusion tensor imaging during recovery from severe traumatic brain injury and relation to clinical outcome: a longitudinal study. Brain.

[bb0205] Singh M., Jeong J., Hwang D., Sungkarat W., Gruen P. (2010). Novel diffusion tensor imaging methodology to detect and quantify injured regions and affected brain pathways in traumatic brain injury. Magn. Reson. Imaging.

[bb0210] Smith D.H., Meaney D.F., Shull W.H. (2003). Diffuse axonal injury in head trauma. J. Head Trauma Rehabil..

[bb0215] Smith S.M. (2002). Fast robust automated brain extraction. Hum. Brain Mapp..

[bb0220] Smith S.M., Jenkinson M., Johansen-Berg H., Rueckert D., Nichols T.E., Mackay C.E., Watkins K.E., Ciccarelli O., Cader M.Z., Matthews P.M., Behrens T.E. (2006). Tract-based spatial statistics: voxelwise analysis of multi-subject diffusion data. NeuroImage.

[bb0225] Smith S.M., Jenkinson M., Woolrich M.W., Beckmann C.F., Behrens T.E., Johansen-Berg H., Bannister P.R., De Luca M., Drobnjak I., Flitney D.E., Niazy R.K., Saunders J., Vickers J., Zhang Y., De Stefano N., Brady J.M., Matthews P.M. (2004). Advances in functional and structural MR image analysis and implementation as FSL. NeuroImage.

[bb0230] Tozer D.J., Chard D.T., Bodini B., Ciccarelli O., Miller D.H., Thompson A.J., Wheeler-Kingshott C.A. (2012). Linking white matter tracts to associated cortical grey matter: a tract extension methodology. NeuroImage.

[bb0235] Traynor C., Heckemann R.A., Hammers A., O'Muircheartaigh J., Crum W.R., Barker G.J., Richardson M.P. (2010). Reproducibility of thalamic segmentation based on probabilistic tractography. NeuroImage.

[bb0240] Wang J.Y., Bakhadirov K., Abdi H., Devous M.D., Marquez de la Plata C.D., Moore C., Madden C.J., Diaz-Arrastia R. (2011). Longitudinal changes of structural connectivity in traumatic axonal injury. Neurology.

[bb0245] Whitnall L., McMillan T.M., Murray G.D., Teasdale G.M. (2006). Disability in young people and adults after head injury: 5–7 year follow up of a prospective cohort study. J. Neurol. Neurosurg. Psychiatry.

[bb0250] Woolrich M.W., Jbabdi S., Patenaude B., Chappell M., Makni S., Behrens T., Beckmann C., Jenkinson M., Smith S.M. (2009). Bayesian analysis of neuroimaging data in FSL. NeuroImage.

[bb0255] Xue R., van Zijl P.C., Crain B.J., Solaiyappan M., Mori S. (1999). *In vivo* three-dimensional reconstruction of rat brain axonal projections by diffusion tensor imaging. Magn. Reson. Med..

[bb0260] Zhang Y., Brady M., Smith S. (2001). Segmentation of brain MR images through a hidden Markov random field model and the expectation–maximization algorithm. IEEE Trans. Med. Imaging.

[bb0265] Zhang Y., Zhang J., Oishi K., Faria A.V., Jiang H., Li X., Akhter K., Rosa-Neto P., Pike G.B., Evans A., Toga A.W., Woods R., Mazziotta J.C., Miller M.I., van Zijl P.C., Mori S. (2010). Atlas-guided tract reconstruction for automated and comprehensive examination of the white matter anatomy. NeuroImage.

