# Supplementary material for: A robust method for investigating thalamic white matter tracts after traumatic brain injury
Source: Neuroimage. 2012 Nov 1;63(2):779–88. doi: 10.1016/j.neuroimage.2012.07.016 (PMC3471070; doi:10.1016/j.neuroimage.2012.07.016)
Supplement: Supplementary Fig. 1 — Skeletons obtained using the 10 subjects from which the template is obtained (row a), the other 11 healthy subjects (row b) and the whole healthy population (c) in three slices in standard space. The skeletons are overlaid in different colors (row d), depicted in yellow, green and blue respectively. The voxels common to all three skeletons are depicted in red. [file mmc1.pdf]

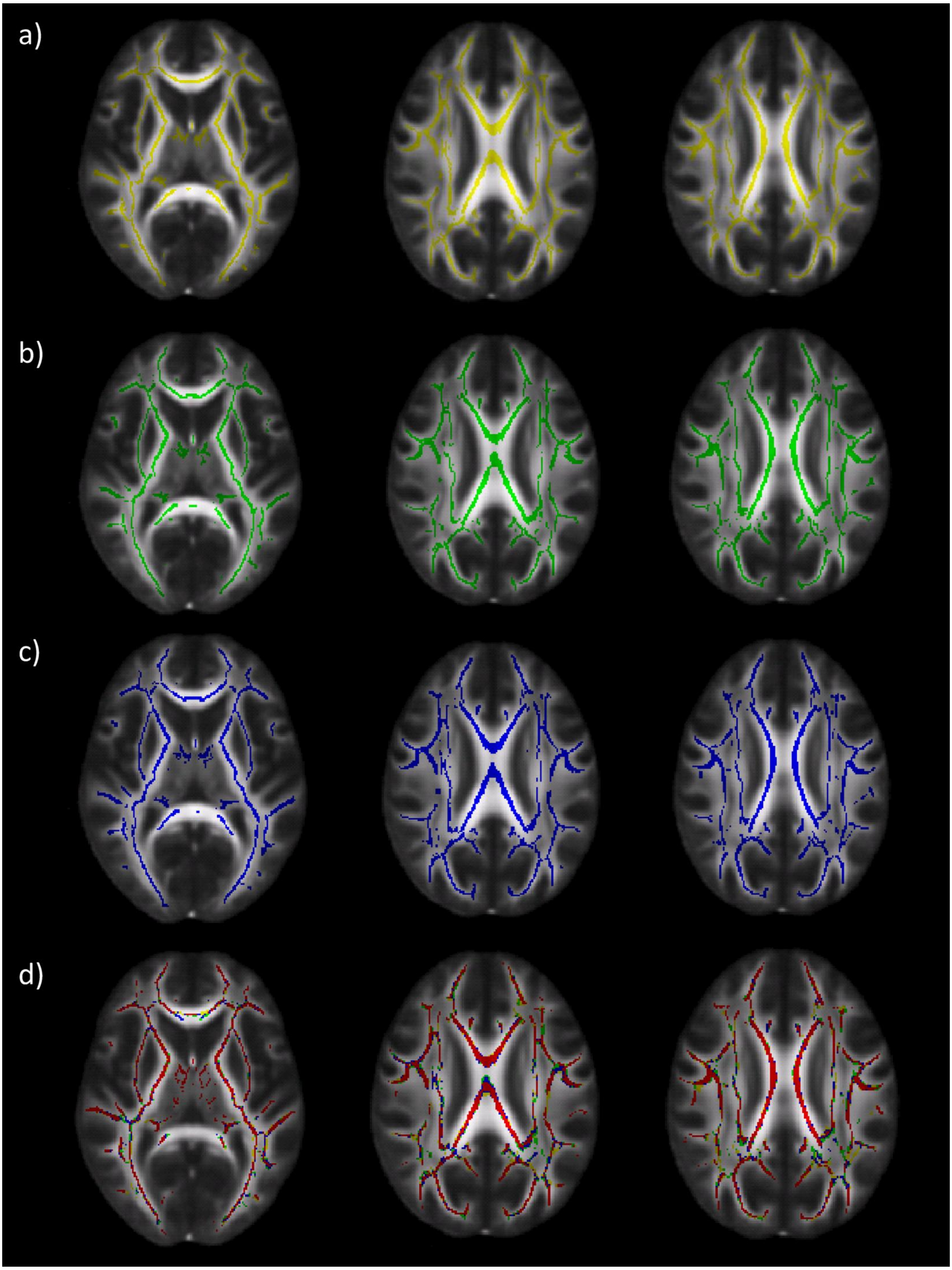

Supplementary Figure 1 - Skeletons obtained using the 10 subject from which the template is obtained (row a), the other 11 healthy subjects (row b) and the whole healthy population (c) in three slices in standard space. The skeleton are overlaid in different colors (row d), depicted in yellow, green and blue respectively. The voxels common to all three skeletons are depicted in red.
