# Supplementary material for: A robust method for investigating thalamic white matter tracts after traumatic brain injury
Source: Neuroimage. 2012 Nov 1;63(2):779–88. doi: 10.1016/j.neuroimage.2012.07.016 (PMC3471070; doi:10.1016/j.neuroimage.2012.07.016)
Supplement: Supplementary Fig. 2 — Boxplots showing the mean percentage of voxels with MD > (mean MD + 3 sd) in patients and in controls (11 subjects unrelated to the template creation process), for all the 10 tracts considered in this study. The percentages of outlying voxels are consistently smaller for healthy controls, as is to be expected (Wilcoxon rank‐sum test, P = 0.05). [file mmc2.pdf]

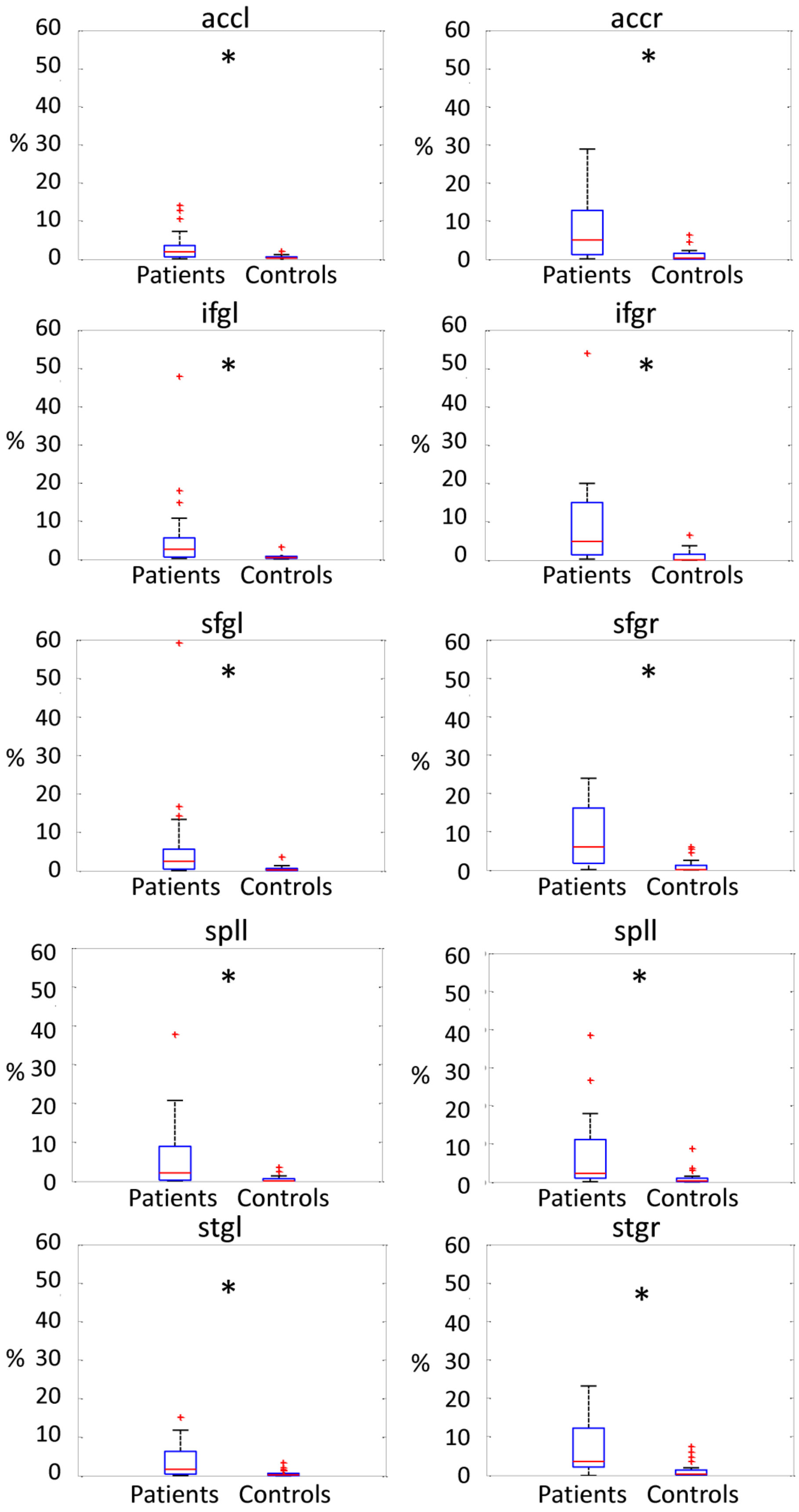

Supplementary Figure 2 - Boxplots showing the mean percentage of voxels with MD > (mean MD + 3sd) in patients and in controls (11 subjects unrelated to the template creation process), for all the 10 tracts considered in this study. The percentages of outlying voxels areconsistently smaller for healthy controls, as is to be expected (Wilcoxon rank-sum test, p=0.05).
